# Feeling ‘Right’ When You Feel Accepted: Emotional Acculturation in Daily Life Interactions With Majority Members

**DOI:** 10.3389/fpsyg.2018.01093

**Published:** 2018-08-03

**Authors:** Alba Jasini, Jozefien De Leersnyder, Batja Mesquita

**Affiliations:** ^1^Center for Social and Cultural Psychology, Faculty of Psychology and Educational Sciences, KU Leuven, Leuven, Belgium; ^2^Programme Group of Social Psychology, Faculty of Social and Behavioural Sciences, University of Amsterdam, Amsterdam, Netherlands

**Keywords:** emotional acculturation, interaction, quality of interaction, intercultural interaction, multiculturalism

## Abstract

When immigrant minority individuals engage in frequent and positive social contact with majority culture members, their emotions become a better fit with the majority norm; the increased fit is called *emotional acculturation*. In the current research, we test the prediction that high-quality interactions with majority others, in which minorities feel accepted, increase the likelihood of emotional fit. We also explore whether this prediction holds true for both positive and negative interactions with majority. To test this prediction, we conducted a 7-day daily diary study with minority students in Belgian middle schools (*N* = 117). Each day, participants reported one positive and one negative interaction at school. They subsequently evaluated each interaction (e.g., felt accepted), assessed their relationship with the interaction partner (e.g., our relationship is important to me), and rated their emotions. Analyses focused on the interactions with Belgian majority interaction partners. Emotional acculturation was computed for positive and negative interactions separately, by calculating the fit between the emotional pattern of the minority student and the average emotional pattern of a sample of majority participants (*N* = 106) who also took part in the daily diary. As predicted, we found higher emotional fit in *positive* interactions when immigrant minorities felt accepted by the interaction partner. In contrast to this finding for positive interactions, emotional fit for negative interactions was higher when minorities felt excluded by the interaction partner. Further analyses on the negative interactions suggested that minority adolescents felt more negative autonomy-promoting emotions (e.g., anger and frustration) when they perceived being excluded. Given that Belgian majority youth feel more autonomy-promoting emotions generally, minorities’ fit with majority patterns was higher. The results confirm our hypothesis that minorities’ fit with majority emotions is contingent on the quality of their interactions with majority, even if in negative interactions, high-quality interactions produced less rather than more emotional fit. Our findings suggest that emotional acculturation is not just a ‘skill’ that minority individuals acquire, but also a response to the ways in which interactions with majority others develop. Inclusive interactions, especially when they are positive, appear to align immigrant minority individuals with the majority norm.

## Introduction

When people experience the normative or ‘right’ emotions of their culture, they report better psychological and relational wellbeing as well as fewer somatic symptoms (e.g., Consedine et al., 2014; De Leersnyder et al., 2014, 2015). Yet, the ‘right’ emotions vary across cultures, in ways that can be understood from cultural differences in the central values and goals (Mesquita and Frijda, 1992; Markus and Kitayama, 1994; Kitayama et al., 2006; Mesquita and Leu, 2007; Mesquita et al., 2016; Tamir et al., 2016; De Leersnyder et al., 2017). For instance, in cultural contexts that highlight concerns about personal independence, autonomy, and self-enhancement, *autonomy-promoting* emotions (e.g., feeling proud or angry) are normative and prevalent. In contrast, in contexts that highlight concerns about social harmony, interdependence with others, and self-criticism, *relatedness-promoting* emotions (e.g., feeling close and ashamed) are both “right" and commonly observed.

These observations raise the important question of what happens when people move to a different culture, with a different set of dominant goals and concerns and, therefore, different ‘typical’ or ‘normative’ patterns of emotion. Will there be a misfit between the emotions of immigrant minority individuals and their new majority culture? And will this emotional misfit influence their wellbeing and relationships in the new culture?

The evidence so far suggests that immigrant minorities may initially experience a misfit, but also that, under the right circumstances, minorities’ emotions will acculturate and come to fit the normative emotions of the majority culture (De Leersnyder et al., 2011; De Leersnyder, 2017). Across several studies with Korean American, Turkish Belgian, and diverse samples of minority youth in Belgium (De Leersnyder et al., 2011; Jasini et al., 2018), we measured emotional acculturation as the fit between an immigrant minority individual’s emotional pattern and the average emotional pattern of the majority group in comparable situations. We found that minorities’ level of emotional fit with the typical majority patterns was higher if they had spent more time in the new country and had more social contacts with majority members.

Specifically, we found higher levels of emotional fit with the majority in minorities who had more and better-quality relationships with majority others. A study with a representative and nation-wide sample of minority and majority youth in Belgium yielded a higher fit with majority emotions for minority youth who reported: (i) more (opportunities for) contact with majority members (Jasini et al., 2018), (ii) more majority friends (Jasini et al., unpublished a), and (iii) less rejection by peers and majority teachers (Jasini et al., unpublished b). In sum, emotional fit with majority patterns of emotions is higher for minority individuals who have a greater number of, and more satisfying, contacts with majority others.

One way of understanding emotional fit with the majority is as ‘a skill’ that immigrant minority individuals learn over the course of high quality contact with majority others. Yet, another way of understanding emotional fit with the majority – and one that is not necessarily mutually exclusive with the first explanation – is that minority individuals tend to have higher fit with the majority culture *if and* *when* the majority emotions are relevant for them in the social situation at hand. This is likely the case when minorities feel part of a majority context: minority individuals who have high quality contact with majority others, will *often* have relatively high fit to majority emotions, just because majority contact makes it more relevant to fit the majority norm. If emotional acculturation is contingent on perceived belonging in the moment, it is better understood as a situated response than as a stable ‘skill.’

In the current paper, we will explore this situated approach to emotional acculturation, and examine the kinds of interactions with majority culture members that predict minorities’ emotional fit. In doing so, we shift attention away from characteristics of the minority individual (i.e., emotional fit as a ‘skill’ of the minority member) toward characteristics of the *interaction* between majority and minority members that afford (or constrain) emotional acculturation (i.e., emotional fit as a ‘state’ afforded by the intercultural interaction). Hence, we will examine situation-level variations in the emotional fit of immigrant minority youth as a function of the quality of their interaction with the majority.

### Emotional Acculturation at the Situation Level

The shift in thinking about minorities’ emotional fit not as an individual difference variable and thus a trait, but as a situation-specific variable and thus a state, parallels research on acculturation attitudes, identities, and values that has found that fit with the new cultural norm may vary according to the particular context (for a review see Arends-Tóth and van de Vijver, 2006). For instance, Turkish-Dutch adults endorsed majority (Dutch) values in the *public* domain, but Turkish values in the private domain (e.g., child-rearing) (Arends-Tóth and van de Vijver, 2003). Similarly, experimental studies with biculturals show that situational cues of the majority or the heritage culture, respectively, activate the matching actions, feelings, and thoughts in biculturals (Hong et al., 2000; De Leersnyder, unpublished doctoral dissertation). Together, these studies yield converging evidence that psychological fit with majority (and heritage) culture varies by situation.

In the current research, we propose that emotional acculturation varies along with minorities’ sense of ‘inclusion’ in the majority culture. An inclusive social interaction with a majority member is one that is characterized by the perception of being both accepted and understood by the interaction partner, and one that is devoid of perceived prejudice and misunderstanding. In such interactions, adopting the majority emotions is expected to be particularly relevant for minorities who seek to reciprocate and maintain their connection with the majority interaction partner. This prediction is consistent with evidence that people mimic and adopt the emotional reactions of others in contexts that highlight the affiliation between them and those others. Increased emotional fit has been found between people who like each other (Parkinson, 2011), who share similar attitudes (McHugo et al., 1991), or who belong to the same group (van der Schalk et al., 2011; Hess and Fischer, 2014). Another reason may be that a smooth interaction encourages minorities to ‘try out’ and practice their majority emotional repertoires, or at the very least, releases inhibitions against it. For all these different reasons, we predict that in inclusive social interactions with majority members, minority members are more likely to feel emotions that fit with the majority norm.

However, with few exceptions, studies suggesting that situations of affiliation between people are associated with increased emotional fit have mainly focused on positive emotions and interactions (Hess and Fischer, 2013; Elfenbein, 2014). It is unclear if a similar association between affiliative negative interactions and emotional fit will occur. We explore this relationship, against two competing predictions. The first is that, even in the context of a negatively valenced encounter, feeling accepted and respected by their majority member may make a safe place for minorities to adopt and perform the culturally promoted emotions. Such finding would concord with previous experimental findings in romantic dyads showing that the relationship quality was positively associated with emotional similarity regardless of the emotional valence of the interaction (Gonzaga et al., 2007). An alternative prediction would be that the quality of majority contact in negative situations is associated with lower emotional fit. This may be possible since minorities may be more likely to react with complementary (i.e., guilt in response to anger) rather than reciprocal negative emotions when they feel included by majority members in negative interactions. In addition, if minorities are often subjected to exclusion by majorities, they may over time learn to respond with majority-like emotions to their majority interaction partners in exclusionist negative interactions. Such a possibility resonates with previous research evidence indicating that behavioral and affective mimicry may help people to address their heightened affiliation needs after experiences of exclusion (Lakin et al., 2008; Cheung et al., 2015; Hühnel et al., 2017). Adopting the majority repertoire of emotions in negative and exclusive interactions may therefore help minorities to meet their threatened affiliation needs and maintain belonging in the majority group.

In sum, we expect that minorities’ emotional fit at the situation level is predicted by their perceptions of being accepted and included by their majority interaction partner.

### Overview of the Current Research

The current research examines variations in emotional fit between minority individuals and the majority at the level of social interactions. We predict that the degree to which minority individuals feel accepted and included will be associated with their *momentary* emotional fit with the majority emotion norms. Specifically, we expect that high perceived inclusion predicts high emotional fit in minorities. In addition, we explore whether this prediction holds true for both positive and negative situations. To examine our hypothesis, we conducted a 7-day diary study with immigrant minority youth in Flanders, Belgium. Each day, participants reported on one positive and one negative emotional interaction they had at school. For each interaction, they rated their emotional experiences as well as the momentary quality of their interaction. At the end of the diary study, participants provided information on the respective interaction partners for each of the episodes reported during the daily diary study.

## Materials and Methods

### Participants

One hundred and seventeen minority youth (44 boys, 73 girls, aged 14 to 19, *M*_Age_ = 15.60) and 106 majority youth (35 boys and 71 girls, aged 13–18, *M*_Age_ = 14.89) from seven secondary schools in Flanders, Belgium participated in the daily diary study^1^. Most of the minority participants were from Moroccan-origin (*N* = 40, 34%) and Turkish-origin (*N* = 21, 18%) families. The other minority participants came from families that originated from around the world, with no country represented by more than 5% of the minority participants (see online Supplementary Materials for further information on these other minorities’ countries of origin). Seventy seven percent of the minority participants were born in Belgium (*N* = 90) and, in response to the question if they had ‘spent most of their lifetime in Belgium,’ 92% answered affirmatively (*N* = 108). Since the present paper focuses on minorities’ emotional fit with the majority culture in interactions with majority members, we primarily used the data coming from minority participants. We only made use of the majority data to compute the majority reference patterns that allowed us to calculate minorities’ emotional fit with the majority culture for specific types of social interactions.

Only 78% (*N* = 91) of the initial minority sample of 117 was included in the final analyses; data from the other participants had to be dropped (see below on case inclusion).

### Procedure

The study collected data from youth attending secondary schools in Flanders, which were selected through a convenience sampling procedure from a pool of schools that had previously participated in the Leuven Children of Immigrants Longitudinal Study (Leuven-CILS), a study with a nationwide sample of schools (Coskan et al., 2012). For the current study, we selected schools with mixed minority-majority student populations (10–60% of the school population were immigrant minority youth). Inclusion of these schools made it possible to reach minority student participants, and also guaranteed that minority and majority students interacted on an everyday basis. Several classes per school were selected for participation in our study.

For each school, we obtained permission from administrators to conduct our study. We also followed an opt-out informed consent procedure for the both students’ parents and the students themselves. To boost the level of participation, we made incentives contingent on the degree of participation. Participants who completed at least one of the questionnaires, received a movie ticket for their participation. Those who completed at least 50%, received an additional 5-Euro voucher. In addition, the class with the highest level of participation within each school won a pizza party at the school premises.

Data collection followed several steps: First, a research team introduced the goals and the procedure of the study during school hours and in the presence of a teacher. At this point, we also distributed informed consent forms for both parents and children. Two weeks later, the research team returned to the classrooms, collected any signed forms of non-consent, and conducted a training session about the online questionnaire. During the training session, participants worked on a trial version of the questionnaire in their school’s computer classrooms, following the research team’s instructions on the questionnaire. Participants also received a booklet with instructions and examples to which they could refer during both the training session and the online questionnaire in the days following. A teacher was present during the training session. At the end of the training session, participants provided the research team with their email addresses.

Participants received the daily diary questionnaire for seven consecutive school days. Each day, minority and majority youth participants started the questionnaire by describing two specific interactions they had had that day at school, and that had made them feel either bad or good. For each interaction, minorities reported information on their interaction partner and on their relationship with this partner, the intensity of their emotions, and their perception of the quality of contact. On different days, the questionnaires were identical in content, but the order of questions differed. On the odd days, participants answered questions about the negative interactions first, followed by questions about the positive interaction; on the even days, they first answered questions on the positive interaction. At the end of the daily diary study, which was the eighth day, participants received a new type of questionnaire, in which they reported on their own cultural background and those of each of the interaction partners in the emotional episodes reported during the previous seven days. We saved this last questionnaire to the end, in order to avoid drawing attention to minority/majority status. Until the very end, participants were unaware, therefore, that our research focus was on minority/majority contact.

Participants completed the questionnaires online, outside of school hours. They received an email at 4 p.m. that invited them to access the link of the questionnaire. At 8 p.m., those who had not yet completed the questionnaire received a reminder.

### Materials

#### Emotional Acculturation

To measure emotional acculturation, we used the Emotional Pattern Questionnaire (EPQ). The EPQ has been developed to measure patterns of emotional experience in well-defined situation types (prompts) (De Leersnyder et al., 2011). The EPQ in this study consisted of prompts that specified context (school) and valence (an interaction in which you felt good/bad). We chose school interactions because schools afford minorities many opportunities for interactions with majority members such as majority classmates, schoolmates and teachers. In addition, interactions reported in the school context would be comparable across minorities and majorities; the latter were the reference group for emotional fit. The prompts of the EPQ in this study read: *Hereafter we ask you to describe one interaction you have had today at your school. Some interactions make you feel bad (good), others make you feel good (bad). Now we will ask you to describe one interaction in which you felt bad (good). If you interacted with more than one person, enter the name of the person who was most important in that situation.*

Each participant received both the negative and the positive prompts. For each prompt, the participants were asked to recall and describe an interaction and give the name of their interaction partner. Then, participants rated the degree to which they had felt a list of emotions during the interaction they had reported. The EPQ contained 13 emotion rating scales, selected to be the highest loading items on one of four emotion factors that emerged from previous research (Jasini et al., 2018). The factors consisted of positive autonomy-promoting emotion (‘*I felt, happy; proud; surprised; elated’*), positive relatedness-promoting emotion (*‘I felt connected; relying upon; respectful’)*, negative autonomy-promoting emotions (‘*I felt frustrated; angry; disappointed’), and* negative relatedness-promoting emotion items were *‘I felt guilty; ashamed; indebted.’* To help us check whether the reported interactions fit the valence prompts, we also included the items ‘*I felt* *good’* and *‘I felt sad.’* All items were rated on 5-point scales (1 = ‘not at all’, 5 = ‘very much’).

To test the equivalence of emotion items across minority and majority groups, we conducted a Simultaneous Component Analysis (Roover et al., 2012) with two clusters (minority and majority) and four hypothetical components (negative autonomy-promoting emotions, negative relatedness-promoting emotions, positive autonomy-promoting emotions and positive relatedness-promoting emotions). We conducted SCA separately for negative and positive interactions. The SCA indicated a two- (rather than a four-) components solution for both minority and majority groups and for both positive and negative types of interactions. Based on the SCA, we excluded surprise, which loaded on different factors both for negative and positive interactions, and for minority and majority participants. Of the remaining twelve emotions, the six negative emotion items loaded systematically on a negative emotion component and the six positive emotion items loaded on a positive emotion component. Emotional fit calculations were based on these 12 items. The factors yielded by the SCA accounted for substantial variation in all types of interactions and in both minority and majority samples (43.87% of the total variance was explained in negative interactions and 54.88% of variance was explained in positive interactions).

For each minority student, we computed two scores of emotional fit with the typical majority patterns of emotion: one for negative, and one for positive interactions. A minority student’s fit was calculated by correlating the pattern of ratings on the 12 different emotions with the average majority ratings on the same emotions in same-valenced interactions (positive versus negative). For instance, to compute a minority student’s emotional fit with the majority culture in the context of a negative interaction, we correlated his or her pattern of emotions reported in that interaction with the average pattern of emotions reported by all the majority participants in negative interactions. The same procedure was followed to compute minorities’ emotional fit in positive interactions. Fit scores (i.e., correlation coefficients) were Fisher transformed before being subjected to further analyses.

#### Who Is the Interaction Partner?

Whether the interaction partner was a minority or majority member was inferred from the cultural background and language spoken at home by the interaction partner, as reported by the minority participant on the last day questionnaire.

#### Quality of Majority Contact

The quality of contact during the social interaction was measured by evaluative statements of the interaction and the interaction partner, as they have been used in other intergroup-research (Shelton et al., 2014; Van Acker et al., 2014; Mallett et al., 2016). These statements gauge such interaction qualities as threat versus safety, and exclusion versus inclusion as well as rejection versus appreciation of the interaction partner. The items were: *I felt anxious; I felt insecure; I felt admiration for the other person; I felt respected by the other person; I felt stressed; I felt understood by the other person; I felt validated by the other person; I felt appreciated by the other person; I felt misunderstood by the other person; I felt left out; I felt abandoned; I felt ignored; I felt that the other person kept me at a distance; My interaction partner had a peculiar way of saying and doing things; The behavior of my interaction partner was appropriate; My interaction partner shares values that are important to me; My interaction partner did not respect my way of thinking.* All items were rated in a 5-point scale (1 = ‘not at all’; 5 = ‘very much’).

An exploratory factor analysis (Extraction Method: Principal Component Analysis; Rotation Method: Varimax) on these items, yielded four factors in the negative interactions (Total Variance explained = 63.90%) and three factors in the positive interactions (Total Variance explained = 58.89%). To extract a comparable factor solution for negative and positive interactions, we computed similar factor analyses but forced the number of components to three for both negative (Total Variance explained = 57.94%) and positive interactions (Total Variance explained = 58.89%). We excluded the items that did not systematically load on the same components across both types of interactions (*I felt stressed; I felt misunderstood by the other person; The behavior of my interaction partner was appropriate)*, and created three scales based on the factors that had emerged: exclusion by majority interaction partner, acceptance by majority interaction partner, and majority interaction partner is different.

##### Exclusion by the majority interaction partner

This scale consisted of six items, capturing minorities’ perceptions and feelings about being excluded by the majority interaction partner (*M* = 2.15, *SD* = 0.93 for negative interactions; *M* = 1.36; *SD* = 0.58 for positive interactions) (α = 0.835 for negative interactions; α = 0.885 for positive interactions). The items were: *I felt anxious; I felt insecure; I felt left out; I felt abandoned; I felt ignored; I felt that the other person distanced me.*

##### Acceptance by the majority interaction partner

This scale consisted of six items, measuring minorities’ perception and feelings about being accepted by the majority interaction partner (*M* = 1.88; *SD* = 0.88 for negative interactions; *M* = 3.47. *SD* = 0.85 for positive interactions) (α = 0.894 for negative interactions, and α = 0.819 for positive interactions). The items were: *I felt respected by the other person; I felt understood by the other person; I felt validated by the other person; I felt appreciated by the other person; I felt admiration for the other person; My interaction partner shares values that are important to me*.

##### Majority interaction partner is different

This scale consisted of two items, measuring minorities’ perception about the majority interaction partner as being peculiar and different from oneself (*M* = 2.87; *SD* = 1.23 for negative interactions; *M* = 1.60; *SD* = 0.80 for positive interactions) [*r*(510) = 0.545, *p* < 0.001 for negative interactions; *r*(489) = 0.427, *p* < 0.001 for positive interactions]. The items were: *My interaction partner had a peculiar way of saying and doing things; My interaction partner did not understand my way of thinking*.

In addition to the momentary quality of interaction measured by the three scales above, we also measured the strength of the relationship between minorities and their majority interaction partner. This particular measure was conceptualized as a more distal measure of the quality of contact with the majority interaction partner. We expected that minority members would experience better quality of contact with close and important than with distant and less important majority interaction partners.

##### Relationship strength

This scale consisted of two items, measuring the strength of the relationship with the majority interaction partner (*M* = 3.80; *SD* = 1.10 for negative interactions; *M* = 2.88; *SD* = 1.36 for positive interactions) [*r*(497) = 0.832, *p* < 001 for negative interactions, and *r*(471) = 0.750, *p* < 0.001 for positive interactions]. The items were: *How close do you feel toward the interaction partner?* and *How important is the interaction partner for you? (1 = ‘not close/important at all’; 2 = ‘not close/important’; 3 = ‘a little bit close/important’; 4 = ‘close/important’; 5 = ‘very close/important’).*

#### Control Variables

We also asked participants to report demographic information about themselves (age, gender) and to describe their relationship with the interaction partner. Descriptive relationship measures were length of acquaintance and frequency of contact.

##### Length of acquaintance

The length of acquaintance with the interaction partners was measured with one question: *How long have you known the interaction partner? (1 = ‘We just met,’ 2 = ‘Less than a week,’ 3 = ‘Less than a month,’ 4 = ‘Less than a year,’ 5 = ‘More than a year’).* Given that most participants knew their interaction partners for a long time, we created a dichotomous variable with two values: 1 = ‘having met less than a year ago’ (i.e., short period of acquaintance: 26.70% in negative interactions; 29.30% in positive interactions) to 2 = ‘having known each other for more than a year’ (i.e., long period of acquaintance: 63.30% in negative interactions; 70.70% in positive interactions).

##### Frequency of contact

The frequency of contact was measured with one question: *How often do you interact with the interaction partner? (1 = ‘Once a day,’ 2 = ‘Once a week,’ 3 = ‘Once a month,’ 4 = ‘Once a year,’ 5 = ‘Almost never’).* Given that participants interacted with their interaction partners relatively often, we created a dichotomous variable with two values: 1 = ‘Once a week or less’ (i.e., infrequently: 46.50% for negative interactions; 31.80% for positive interactions), and 2 = ‘Once a day’ (i.e., very frequently; 53.50% for negative interactions; 68.20% for positive interactions).

There was no multicollinearity between measures of interaction quality and the relationship measures. The correlations between these measures were moderate at most (see **Table 1**).

**Table 1:** Correlations between measures of quality of interaction and relationship with the majority interaction partner.

	Exclusion by majority partner	Acceptance by majority partner	Majority partner is different	Relationship strength	Frequency of contact	Length of acquaintance
Exclusion by majority partner	–	0.040	0.227^∗∗∗^	0.177^∗∗^	−0.008	0.020
Acceptance by majority partner	−0.324^∗∗∗^	–	−0.496^∗∗∗^	0.364^∗∗∗^	0.069	0.032
Majority partner is different	0.512^∗∗∗^	−0.286^∗∗∗^	–	−0.265^∗∗∗^	−0.059	−0.056
Relationship strength	−0.198^∗∗^	0.480^∗∗∗^	−0.149^∗^	–	0.485^∗∗∗^	0.257^∗∗∗^
Frequency of contact	−0.095	0.178^∗∗^	−0.050	0.457^∗∗∗^	–	0.202^∗∗^
Length of acquaintance	−0.133^∗∗^	0.092	−0.159^∗^	0.417^∗∗∗^	0.207^∗∗^	–

*The upper part of the table contains the correlation values found in the negative interactions data. The lower part of the table contains the correlation values found in the positive interactions data. Spearman’s rho correlation for non-parametric data (given that we have two categorical variables). ^∗^p < 0.05, ^∗∗^p < 0.01, ^∗∗∗^p < 0.001 (2-tailed).*

### Cases Included

Reported interactions were only included in the analyses when they matched the prompt. Two research assistants who spoke the language of the participants (Dutch) evaluated this match, following a standardized scheme. First, they checked this match by carefully reading the written description of the interaction. For instance, a situation was coded as matching the positive valence prompts when the reported situation clearly described a positive situation. Second, we compared the valence of the prompt with the reported intensity on the emotion items ‘*I felt* *good’* and *‘I felt sad.’* Situations matched with positive valence prompts if ratings on *‘I felt good’* were higher than ratings on ‘*I felt sad’*; the reverse was true for the match of situations with the negative valence prompts. For cases, when the first and second step led to inconsistent coding of whether the situation matches the prompt, we compared in a third step the valence of the prompt with the reported intensities of ‘*I felt angry’* and ‘*I felt happy.’* Situations matched with positive valence prompts if ratings on *‘I felt happy’* were higher than ratings on ‘*I felt angry’*; the reverse was true for the match of situations with the negative valence prompts. Based on the third step comparison, we made a final judgment on whether the situation reported instigated emotions that fit the valence prompts or not. If by these comparisons, the reported interactions did not match the valence prompts, they were omitted from further data analyses. We also excluded cases, when the reported interactions did not take place at school^2^. Following these procedures, left us with 504 (75.79% of 665) negative interactions and 480 (72.18% of 665) positive interactions that matched the prompts.

We further reduced the number of interactions included in our analyses, by selecting only interactions with *majority* others. Based on the information on the minority/majority background of the interaction partner, we limited our analyses to those cases where minority students had reported interactions with Belgian majority members [*N* (294, 58.33% of the prompt-congruent negative interactions; N (273, 56.87% of the prompt-congruent positive interactions].

### Hypothesis Testing

Taking into account the nested nature of the data (interactions nested within participants), we tested our hypotheses via multilevel regression analyses. We estimated two-level random-intercept models using MLwiN 2.29. Overall, log-likelihood ratio tests confirmed that emotional fit scores varied significantly at the level of interactions (level 1) as well as the level of participants (level 2). The overall variation in emotional fit in negative interactions was 77% at the interaction level and 23% at the person level. The overall variation in emotional fit in positive interactions was 56% at the interaction level and 44% at the person level. This is first evidence that emotional fit varies at the level of the situation, in addition to being a person-level variable.

We estimated regression models for negative and positive types of interaction separately. Each time, we estimated three models: a null intercept model, a model with controls (age, centered around the grand mean; gender, with ‘*boy*’ as reference category; frequency of contact with the majority interaction partner, with ‘*seeing each other less than once a week*’ as reference category; length of acquaintance with the majority interaction partner, with ‘*knowing each other for less than a year*’ as reference category), and a model where we entered the control measures and the predictor of interest.

Our hypotheses were supported when (i) the more complex models (model with the controls and predictor of interest) better fit the data than the simpler models (model with the controls) and (ii) the associations as defined by the standardized regression coefficients were statistically significant and in the expected direction. We estimated all models using a Maximum Likelihood approach (IGLS or iterative generalized least squares). To test whether each model better fit the data than the respective simpler model, we used the Likelihood Ratio Test (Snijders and Bosker, 2012), which estimates whether the difference between the fit indicators of each model (-2 loglikelihood) is different from zero; the difference is χ^2^ distributed. To test the significance of the effect estimates (the fixed effects in the model), we used two-tailed *t*-tests (significance level: *p* = 0.05).

In all analyses, we followed a list-wise deletion procedure for cases where data on at least one of the variables of interest was missing.

## Results

### Hypothesis: Minority’s Emotional Fit With the Majority Norm Is Positively Predicted by the Quality of the Social Interaction

#### Positive Situations

To test our hypothesis in positive situations, we conducted a series of multilevel regression models with data on positive interactions only. We estimated separate models for each measure of quality of social interaction (see **Table 2**). In separate models, we entered the predictors *exclusion by the majority interaction partner* (Model 1), *acceptance by the majority interaction partner* (Model 2), *the majority interaction partner is different* (Model 3), and *strength of relationship with the majority interaction partner* (Model 4), while controlling for both person-related factors (*age* and *gender* of the minority participant) and factors describing the relationship with the interaction partner (*frequency of contact with the majority interaction partner* and *length of acquaintance with the majority interaction partner*). All models fit the data better than both the null models and the models with only the controls.

**Table 2:** Minorities’ emotional fit predicted by the quality of positive interactions.

	Null model	Null model (with controls)	Model 1	Model 2	Model 3	Model 4
**Fixed part**						
Intercept	1.443 (0.069)^∗∗∗^	0.985 (0.133)^∗∗∗^	1.029 (0.115)^∗∗∗^	1.070 (0.113)^∗∗∗^	1.051 (0.125)^∗∗∗^	1.315 (0.140)^∗∗∗^
Age		−0.058 (0.062)	−0.074 (0.052)	−0.032 (0.051)	−0.059 (0.058)	−0.066 (0.058)
Gender		0.220 (0.134)	0.088 (0.113)	0.125 (0.110)	0.130 (0.126)	0.160 (0.125)
Frequency of interactions		0.270 (0.090)^∗∗^	0.329 (0.083)^∗∗∗^	0.195 (0.082)^∗^	0.274 (0.087)^∗∗^	0.073 (0.095)
Length of acquaintance		0.238 (0.098)^∗^	0.163 (0.089)^†^	0.224 (0.088)^∗^	0.195 (0.094)^∗^	0.063 (0.100)
Exclusion by majority partner			−0.592 (0.076)^∗∗∗^			
Acceptance by majority partner				0.414 (0.050)^∗∗∗^		
Majority partner is different					−0.264 (0.052)^∗∗∗^	
Relationship strength						0.240 (0.047)^∗∗∗^
**Random part**						
Level: Participant	0.262 (0.061)	0.226 (0.054)	0.138 (0.037)	0.130 (0.036)	0.187 (0.047)	0.187 (0.047)
Level: Interaction	0.336 (0.035)	0.301 (0.032)	0.264 (0.028)	0.261 (0.027)	0.282 (0.030)	0.281 (0.030)
–2^∗^loglikelihood:	567.657	523.290	467.045	464.530	497.808	496.384
Units: Participant	85	85	85	85	85	85
Units: Interaction	269	262	260	262	261	261

*^†^p < 0.10, ^∗^p < 0.05, ^∗∗^p < 0.01, ^∗∗∗^p < 0.001 (2-tailed).*

In line with our hypothesis, in positive interactions, minorities’ emotional fit was positively associated with feelings of being accepted by the majority partner in interaction [model 2: Δ-2LL(1) = 58.76, *p* < 0.001; *t*(257) = 8.28, *p* < 0.001] and the strength of their relationship [model 4: Δ-2LL(1) = 26.91, *p* < 0.001; *t*(256) = 5.11, *p* < 0.001]. In addition, minorities’ emotional fit was negatively associated with feelings of being excluded by the majority partner [model 1: Δ-2LL(1) = 56.25, *p* < 0.001; *t*(255) = −7.79, *p* < 0.001] and perceptions of the majority interaction partner as different from oneself [model 3: Δ-2LL(1) = 25.48, *p* < 0.001; *t*(256) = −5.07, *p* < 0.01] (please see **Table 2** for the full results). Thus, congruent with our hypothesis, in positive interactions with majority, the more minorities perceived the quality of their interactions with the majority as positive, the more they fitted emotionally with the normative majority patterns.

#### Negative Situations

To test our hypothesis in negative situations, we conducted a series of multilevel regression models with data on negative interactions only. Similar to the analyses on positive interactions, we estimated separate models for each measure of the quality of social interaction, while controlling for both demographic factors (*age* and *gender* of the minority participant) and factors describing the relationship with the interaction partner (*frequency of contact with the majority interaction partner* and *length of acquaintance with the majority interaction partner*): *exclusion by the majority interaction partner* (Model 1), *acceptance by the majority interaction partner* (Model 2), *the majority interaction partner is different* (Model 3), and *strength of relationship with the majority interaction partner* (Model 4). Again, all models fit the data better than both the null models and models with only the controls.

However, contrary to our hypothesis, we found that minorities’ emotional fit was *negatively* (instead of positively) related to feelings of inclusion in negative interactions. Concretely, both the perceived acceptance by the majority interaction partner [model 2: Δ-2LL(1) = 47.34, *p* < 0.001; *t*(272) = −7.31, *p* < 0.001], and the strength of the relationship with the majority interaction partner [model 4: Δ-2LL(1) = 8.50, *p* < 0.001; *t*(272) = 2.92, *p* < 0.01] were negatively associated with the minority individual’s level of emotional fit with the majority. Moreover, emotional fit in the negative interactions was *positively* (instead of negatively) related to perceptions both of exclusion by the majority interaction partner [model 1: Δ-2LL(1) = 8.79, *p* < 0.01; *t*(272) = 2.97, *p* < 0.01] and of the majority interaction partner as *different* [model 3: Δ-2LL(1) = 21.17, *p* < 0.001; *t*(272) = 4.67, *p* < 0.001] (please see **Table 3** for the full results). Different from our findings for positive interactions, low quality of interactions with majority in negative interactions predicts emotional fit with the normative majority patterns.

**Table 3:** Minorities’ emotional fit predicted by the quality of negative interactions.

	Null model	Null model (with controls)	Model 1	Model 2	Model 3	Model 4
**Fixed part**						
Intercept	0.642 (0.039)^∗∗∗^	0.657 (0.080)^∗∗∗^	0.659 (0.080)^∗∗∗^	0.621 (0.073)^∗∗∗^	0.653 (0.079)^∗∗∗^	0.570 (0.084)^∗∗∗^
Age		−0.045 (0.035)	−0.039 (0.035)	−0.042 (0.032)	−0.041 (0.035)	−0.048 (0.034)
Gender		0.029 (0.082)	0.036 (0.082)	0.027 (0.075)	−0.009 (0.082)	0.036 (0.081)
Frequency of interaction		−0.182 (0.063)^∗∗^	−0.181 (0.062)^∗∗^	−0.114 (0.059)^†^	−0.167 (0.061)^∗∗^	−0.100 (0.068)
Length of acquaintance		0.075 (0.064)	0.068 (0.063)	0.080 (0.059)	0.094 (0.062)	0.110 (0.065)^†^
Exclusion by majority partner			0.095 (0.032)^∗∗^			
Acceptance by majority partner				−0.212 (0.029)^∗∗∗^		
Majority partner is different					0.112 (0.024)^∗∗∗^	
Relationship strength						−0.076 (0.026)^∗∗^
**Random part**						
Level: Participant	0.060 (0.020)	0.069 (0.021)	0.070 (0.021)	0.055 (0.017)	0.073 (0.021)	0.066 (0.020)
Level: Interaction	0.199 (0.020)	0.183 (0.019)	0.175 (0.018)	0.155 (0.016)	0.165 (0.017)	0.178 (0.018)
–2^∗^loglikelihood:	404.401	380.379	371.587	333.035	359.205	371.881
Units: Participant	91	90	90	90	90	90
Units: Interaction	284	277	277	277	277	277

*^†^p < 0.10, ^∗∗^p < 0.01, ^∗∗∗^p < 0.001 (2-tailed).*

## Discussion

The present research aimed to show that minorities’ emotional fit varies depending on the characteristics of everyday social exchanges with majorities. This means that emotional fit is more than a personal skill acquired by minority individuals over time, but that it is contingent on everyday fluctuations in the quality of interactions. In line with our expectations, the results show for the first time that minorities’ emotional fit with the majority culture is predicted by minorities’ majority contact even at a momentary and situational level. In addition, it highlights the important role of the quality of social interactions in minorities’ emotional fit, even after controlling for the degree to which minority individuals had previous contact with their majority interaction partners. In sum, these findings suggest that minorities’ emotional acculturation is a complex process of learning and adjustment to the majority culture: while acquiring the capacity to experience culturally congruent emotions through culture exposure and frequent majority contact, minorities adjust their emotional experiences in daily interactions with majority depending on the dynamics and quality of their interactions.

Specifically, our findings show that minorities’ emotional fit at the level of situation, is not always positively associated with the quality of interaction. We expected that in interactions with majority, minorities who felt accepted and understood by the majority interaction partner, would experience emotions closer to those experiences by the majority. This would be the case, because majority emotions become relevant during this momentary connection with the majority interaction partner. Alternatively, these inclusive interactions may afford minorities a ‘safe’ context to practice their majority emotions without feeling any prejudice against them for trying. However, the expectation that good quality of interaction is associated with higher emotional fit, was met only in the context of positive interactions with majority. In negative interactions, minorities had higher fit when they felt excluded. Below, we take the opportunity to further explore why poor quality of negative interactions may be conducive to high emotional fit with the majority culture.

### Negatively Valenced Interactions

There are several reasons why we may have found a negative instead of a positive link between minorities’ emotional fit and their quality of interaction in negative situations with majority members. Here, we elaborate on two potential explanations and report on exploratory *post hoc* analyses to test these explanations with the data available in our study. Full results on these analyses can be found in the Online Supplementary Materials (OSM).

One possible explanation for the lower emotional fit is that minority individuals who are in a close relationship with majority members have emotions complementary to anger, rather than reciprocating anger. This is consistent with insights that (a) dyadic interaction partners often have complementary emotions in response to each other’s emotions (i.e., guilt in response to anger) (Keltner and Haidt, 1999); and (b) complementary emotions benefit the relationship between interaction partners, as they reduce the intensity of the negative emotions communicated in the dyad, while simultaneously leading to conflict avoidance, soothing and affiliation (Gottman et al., 1998; Morris and Keltner, 2000; Van Kleef et al., 2008; Lelieveld et al., 2012). Furthermore, studies with romantic couples have shown that communally oriented partners mimic their partners’ expressions of anger less, whereas exchange oriented (‘tit-for-tat’) partners mimic each other’s anger more (Häfner and IJzerman, 2011). Communally oriented partners pay attention to each other’s needs, and can therefore be said to have a closer relationship than exchange oriented partner who are primarily focused on their own needs. Together, studies on complementary emotions suggest that minorities may be less inclined to reciprocate high intensity anger emotions in the context of an inclusive and accepting interaction with a (close) majority member, due to minorities’ tendency to experience emotions that complement majorities’ emotions in such interaction (e.g., responding with respect rather than anger). The negative emotional interaction may thus be characterized by less emotional fit.

In this vein, we explored the following explanatory mechanism: a good quality of negative interactions predicts a lower emotional fit in minorities because such interactions trigger a high intensity of those emotions that protect rather than threaten the momentary connection with majority (e.g., positive relatedness-promoting emotions such as respect and trust). In contrast, a poor quality of negative interactions predicts a higher emotional fit in minorities because such interactions trigger a high intensity of those emotions that help them reciprocate the negative treatment of the majority interaction partner (e.g., negative autonomy-promoting emotions such as anger and frustration). We believe that this may be especially the case in our study, given that the Belgian majority culture highlights concerns of maintaining personal autonomy (Schwartz, 2006; Boiger et al., 2013), and promotes primarily autonomy-promoting emotions. Thus, on the one hand, experiencing high intensity negative autonomy-promoting emotions when feeling excluded would contribute to a high emotional fit with the majority culture in immigrant minorities. On the other hand, experiencing high intensity of relatedness-promoting emotions in negative interactions, would contribute to a deviation from the typical Belgian majority emotional patterns.

We performed a series of mediation analyses exploring if the intensity of different types of emotions could explain the link between each of our indices of interaction quality. For these analyses, we classified the emotion items in four different categories: negative autonomy-promoting emotions (anger, frustration, and disappointment), positive autonomy-promoting emotions (happiness, elation, and pride), negative relatedness-promoting emotions (shame, guilt, and indebtedness), and positive relatedness-promoting emotions (closeness, trust, and respect) and computed four new variables denoting the mean intensity of each category. Then, we performed separate mediation analyses for each indicator of contact quality (exclusion by the majority interaction partner, acceptance by the majority interaction partner, the majority interaction partner is different, strength of relationship with the interaction partner) and each category of emotions; we did so respecting the multilevel structure of our data and by following the procedure proposed by Krull and MacKinnon (2001).

These analyses revealed that the negative associations between the quality of interaction predictors and minorities’ emotional fit were in general mediated by mean intensities of different categories of emotions. However, the direction of the association changed in the case of the following two mediation analyses. First, when the negative autonomy-promoting emotions variable (i.e., anger) was considered as a mediator in the association between *exclusion by the majority interaction partner* and minorities’ emotional fit, this association was no longer positive but negative: the more they felt excluded by the majority interaction partner in negative interactions, the lower their emotional fit. Second, when the negative relatedness-promoting emotions variable (i.e., closeness) was considered as a mediator in the association between *strength of relationship* and minorities’ emotional fit, this association was no longer negative but positive: the stronger minorities perceived the bond with the interaction partner in negative interactions, the higher their emotional fit (see Supplementary Tables S1–S4, pages 4–7). These findings indicate that the high levels of emotional fit in highly exclusionist negative interactions are especially explained by minorities’ experience of intense negative autonomy-promoting emotions. In a similar vein, the low levels of emotional fit in highly accepting negative interactions are especially explained by minorities’ experience of intense positive relatedness-promoting emotions.

A second potential explanation is that minorities may have lower emotional fit with the majority in negative, high quality interactions because their emotional patterns are more culture-specific in high quality than in low quality negative interactions. Specifically, it is plausible that in negative interactions characterized by high quality, the minority participants in this study experienced more relatedness-promoting emotions (i.e., shame, respect) than their Belgian majority peers, since they mainly originated from “interdependent” cultures where concerns about maintaining relatedness and closeness with others are central. In contrast, the Belgian majority peers would rather experience autonomy-promoting emotions (i.e., anger, pride) in these situations. Differences in the intensities of these emotions between minorities and majorities’ emotional experiences may then explain minorities’ deviation from majorities’ normative pattern, and thus their misfit in these negative situations.

To test this explanation, we conducted a series of multilevel regression analyses with both minority and majority data, focusing only on the negative interactions characterized by high quality of interaction. Again, we classified the emotion items in four different categories: negative autonomy-promoting emotions (anger, frustration, and disappointment), positive autonomy-promoting emotions (happiness, elation, and pride), negative relatedness-promoting emotions (shame, guilt, and indebtedness), and positive relatedness-promoting emotions (closeness, trust, and respect) and computed four new variables denoting the mean intensity of each category. We defined the group of high quality interactions by conducting a median split on each interaction quality variable. In separate regression models, we included the majority status variable as a predictor of participants’ intensity of each emotion category. The analyses showed that in high quality negative interactions, minority and majority youth did not differ in their mean emotional intensities (see Supplementary Tables S5–S8, pages 8–10). These findings suggest that the low levels of minorities’ emotional fit in highly inclusive interactions with majority are not explained by differences in emotional intensities between minority and majority youth.

In sum, we found that the negative (instead of hypothesized positive) association between the quality of interactions and minorities’ emotional fit in negative interactions is driven by the content of the emotional patterns triggered in such situations, and more specifically the intensity of negative autonomy-promoting emotions and positive relatedness-promoting emotions.

### New Insights Into the Process of Emotional Acculturation

The present study contributes to research on emotional acculturation in several important ways. First, it suggests that minorities’ emotional fit is a contextualized process of social adjustment. Our findings show that in the context of interactions with majority members, minorities’ emotional fit is jointly construed in the situation by the dynamics of their communication and interaction. Locating the emotional fit at the micro-level of social interactions allows us therefore to conceptualize this process of emotional alignment not only as a chronic trait of the minority individual, but also as a temporary state of the interactions. Thus, similar to the processes of emotional mimicry and emotional contagion (Hess and Fischer, 2013; Elfenbein, 2014), sharing similar patterns of emotional experiences with majority members is a contextualized process.

Second, the current research findings concur with previous evidence suggesting that acculturation is not a linear process – acculturated traits may be flexible and malleable. Thus, similar to minorities’ cultural affiliations (Doucerain et al., 2013), adoption of cultural norms (Arends-Tóth and van de Vijver, 2003), and tendencies to interpret images or human actions (Hong et al., 2000), minorities’ emotional fit is also sensitive to the characteristics of the particular socio-cultural context. Furthermore, the current findings add to the previous evidence showing that minorities’ emotional fit is higher in contexts that foreground the majority culture rather than the heritage culture (De Leersnyder, unpublished doctoral dissertation). However, our research goes beyond these findings by adding another layer: acculturated traits not only flexibly attune to the majority context when this is foregrounded, but the quality of interaction in that majority context matters as well. In addition, the current work extends previous acculturation studies by showing that minorities’ momentary affiliation with the majority culture is not only tied to the ‘physical’ conditions of the interaction (such as the language of conversation, the type of social activity, or the spoken language and culture of their interlocutor; Doucerain et al., 2013), but most importantly it is tied to the qualitative features of the interaction experience.

Finally, the current study also goes beyond previous research on emotional similarity in dyadic relationships (Anderson et al., 2003), as it shifts the focus from similarity at the level of the dyad to a momentary similarity between minority individuals’ emotions and those that are normative in the culture of their interaction partners. In a similar vein to the dyadic relationships research, the current research was based on the assumption that connection and sense of belonging go hand in hand with sharing similar experiences. As such, our findings on momentary emotional fit in negative interactions echoed findings on emotional similarity in dyads, which indicate that partners in close and happy romantic relationships show low emotional congruence in negative interactions (Gottman et al., 1998). This may be due to the fact that in negative interactions, incongruence in emotions benefits couples’ relationship more than congruence. Finally, the current study extends previous work on emotional similarity in dyads, as it shows that the quality of interactions is important for minorities’ emotional fit regardless of the length of acquaintance time and the frequency of interactions with the interaction partner.

### Limitations and Future Directions

Our study provides important evidence on the mutual constitution of minorities’ momentary emotional fit and the quality of contact with majority individuals. However, the study has some limitations that should be addressed in further research. One such limitation is the cross-sectional nature of the study, which makes it difficult to accurately interpret the causal direction of the association. Is it the quality of the interaction that contributes to minorities’ emotional fit in the moment? Or is it the experience of sharing emotions with majority members that predicts minorities’ perceived quality of the interaction? Even though in the present study, we mainly explained the findings in line with the first interpretation, the opposite direction may also be true: the extent to which minorities share the majority patterns of emotions leads to a better or worse quality of majority contact. The present study is also consistent with mimicry research, showing that the need for affiliation and belongingness is conducive to mimicry but also that mimicry benefits the mutual liking and social bonding between people (Chartrand and Bargh, 1999; Lakin et al., 2003, 2008; Lakin and Chartrand, 2005; Hess and Fischer, 2013; Hühnel et al., 2017). In line with this perspective, when minorities in positive social situations have similar emotional experiences to those that are typical of majorities, this may benefit their connection to the majority member with whom they interact – majority members may find them more similar to themselves, and thus be less inclined to exclude them. On the other hand, when minorities display culturally congruent emotions in negative social situations, this may bring negative consequences for the social bond between minority individuals and their majority interaction partners. In such situations majorities may want to distinguish themselves from the minority individual, and therefore have a higher tendency to exclude him or her. Future experimental studies may help to identify the causal direction in the associations between emotional fit and quality of interaction.

In addition, the current study did not shed light on the processes throughout which minorities come to experience majority emotions in social interactions with majority. One possible direction would be that the repertoire of majority emotions is automatically activated in contexts of positive and inclusive interactions with majority, since such interactions may make minorities feel part of the majority context and thus foreground the majority culture. Another possibility would be that certain goals are triggered in interactions with majority, which may motivate minorities to experience majority emotions. Such goals are the goals to affiliate and belong. Indeed, we can expect that minority participants would be generally motivated to affiliate and belong (Baumeister and Leary, 1995), but even more so when they interact with majority members they know well and interact on a frequent basis. As such, the quality of interactions may touch upon minorities’ needs and activate the goals to affiliate and belong, which in turn may lead minorities to experience majority emotions. This direction would help explain why we find a negative association between quality of interaction and minorities’ emotional fit in negative interactions. Indeed, recent research has shown that emotional contagion and mimicry does not only occur in affiliative and cooperative contexts but also occurs as *a response* to being excluded. Upon exclusion, people may show a tendency for behavioral and affective mimicry to satisfy their heightened need for affiliation and acceptance (Lakin et al., 2008; Hess and Fischer, 2013; Hühnel et al., 2017). Finally, other types of goals and concerns may become important in social interactions and influence minorities’ momentary emotional fit with the majority culture, such as concerns about maintaining the autonomy or relatedness with others, the need to disambiguate the emotional event, or the need to gain control over the situation. Previous research has indeed shown that activation of interdependent self-construals leads to a higher tendency to mimic each other (Van Baaren et al., 2003). Similarly, mimicry may increase when interaction partners need to make sense of a complex situation and thus rely on the other person’s emotional expression (Manstead and Fischer, 2001). Future research may want to test directly whether the quality of interactions predicts minorities’ emotional fit because it automatically activates majority emotions or triggers minorities’ goals of affiliation and belongingness or other social goals.

Moreover, in the current study we did not examine the possibility that emotional fit in negative interactions is higher upon *exclusion* because minority individuals are familiar with these types of interactions. There is ample evidence that immigrant minority youth experience discrimination, exclusion, and rejection by majority peers on a frequent basis. This may contribute to minorities more easily disambiguating interactions with majority in which they feel more rejected and excluded compared to more accepted and included. In such situations, they may find it easier to decode the signals sent by the majority partner and react with a reciprocating emotional pattern that fits the ‘emotional language’ of their partner. A preliminary inspection of the negative interactions data in our research hints at this possibility: in general there was a lower variation in minorities’ emotional fit in highly exclusive negative interactions than in more inclusive negative interactions. However, further research is needed to investigate this issue directly.

Finally, we have failed to capture some other sources of emotional fit. At the situational level, we do not know whether some common event contributed to emotional fit. The occurrence of common events increases the chances that minorities and majorities appraise the meaning of such event from the same vantage point (Elfenbein, 2014). In contrast, in cases where the behaviors of the majority individual are the stimulus for minorities’ emotional experiences, minorities may be more inclined to experience complementary than similar emotions. For instance, in interactions where majority members react with anger and disappointment toward minority individuals, minorities may respond to such treatment with shame and guilt, especially in the context of a close relationship. Experiencing complementary emotions in such case would lead to emotional misfit.

### Conclusion

The present research showed that minorities’ fit with majority emotions varies as a function of the quality of the majority contact. Specifically, we found higher emotional fit when immigrant minorities felt included, accepted by and close to the majority interaction partner in positive situations, and when they felt excluded, rejected by and distant from the majority interaction partner in negative situations. Further analyses on the negative situations suggested that minority adolescents experienced more culturally congruent patterns of emotions in interactions that made them feel excluded and rejected, due to high intensities of negative autonomy-promoting emotions such as anger and frustration experienced in such situations. Overall these findings highlight the role of social situations for minorities’ emotional fit.

## Ethics Statement

This study was carried out in accordance with the recommendations of the Social and Societal Ethics Committee (SMEC, University of Leuven). The protocol was approved by SMEC. We obtained consent from the youth participants and their parents via opt-out consent forms. Such procedure was necessary given that in previous research with immigrant minority youth in Belgium, opt-in procedures had resulted in high levels of drop-out.

## Author Contributions

AJ organized the database, performed the statistical analysis, and wrote the first draft of the manuscript. All authors contributed to the conception, design of the study, wrote sections of the manuscript, contributed to manuscript revision, read, and approved the submitted version.

## Conflict of Interest Statement

The authors declare that the research was conducted in the absence of any commercial or financial relationships that could be construed as a potential conflict of interest.

## Footnotes

1 The number of students entering the study was larger, but we only included participants who had completed the last day questionnaire, which contained the questions on the demographics. As such, it is impossible to compare whether the demographics for the incomplete data are different than for the complete data.

2 In coding the interactions, we kept a broad definition of what constitutes an interaction. Thus, we considered as valid data also those related to situations where participants did not clearly describe instances of interactions. One such situation was for instance ‘*I had a class today with teacher x*,’ in which the participant named *teacher x* as the interaction partner. We decided to consider these cases assuming that in line with the prompts, relevant interactions have taken place in such situations. However, we checked whether the findings would hold if we excluded these less clear cases of interactions, and the data analyses yielded similar patterns of associations to those found and reported in this paper.
