# Supplementary material for: Feeling ‘Right’ When You Feel Accepted: Emotional Acculturation in Daily Life Interactions With Majority Members
Source: Front Psychol. 2018 Aug 3;9:1093. doi: 10.3389/fpsyg.2018.01093 (PMC6085598; doi:10.3389/fpsyg.2018.01093)
Supplement: Supplementary file 1 [file Data_Sheet_1.pdf]

SUPPLEMENTARY MATERIAL

Feeling 'Right' When You Feel Accepted: Emotional Acculturation in Daily Life Interactions With Majority Members

Alba Jasini, Jozefien De Leersnyder, & Batja Mesquita

1 Countries of origin of minority participants

Whereas most of the minority participants originated from Morocco (N = 40, 34%) and Turkey (N = 21, 18%), the other minority participants originated from countries around the world, such as The Netherlands (N = 7, 6%), Germany (N = 6, 5%), Congo (N = 5, 4%), Italy (N = 5, 4%), The Philippines (N = 3, 2.5%), France (N = 3, 2.5%), Kosovo (N = 2, 2%), and Switzerland (N = 2, 2%) (the other countries represented by one participant each (N =19, 16%) were Poland, Senegal, Ecuador, Cambodian, Romania, Ethiopia, Austria, Greece, Portugal, Lebanon, China, Thailand, Guinea, Ghana, Russia, Armenia, Afghanistan, Canada, Somalia). Four minority participants did not indicate their country of origin (N = 4, 3%).

2 Exploratory analyses on negative interactions

2.1 Is the association between interaction quality and emotional fit mediated by emotional intensities?

To examine this question, we conducted a series of mediation analyses on the negative interactions data. For these analyses, we classified the emotion items in four different categories: negative autonomy-promoting emotions (anger, frustration, and disappointment), positive autonomy-promoting emotions (happiness, elation, and pride), negative relatedness-promoting emotions (shame, guilt, and indebtedness), and positive relatedness-promoting emotions (closeness, trust, and respect) and computed four new variables denoting the mean intensity of each category. Then, we performed a series of mediation analyses exploring if the intensity of each category of emotions could explain the link between each of our indices of relationship quality (exclusion by the majority interaction partner, acceptance by the majority interaction partner, the majority interaction partner is different, and strength of relationship with the interaction partner) and emotional fit; we did so respecting the multilevel structure of our data and by following the procedure proposed by Krull and MacKinnon (2001).

To estimate the mediated relationships in a multilevel model, we followed these steps. In the first step, we regressed each mediator (the emotion intensity variables) separately on each variable of quality of interaction. In the second step, we regressed minorities' emotional fit in negative interactions on each mediator. Finally, in the third step, in single regression analyses, we regressed minorities' emotional fit on each of the interaction quality variables on the one hand and the mediators on the other hand. In every step, we also included the four control variables: age, gender, length of acquaintance, and frequency of contact. To maintain sufficient power for the given sample size, we conducted separate mediation analyses for each type of interaction quality and each mediator. We concluded that a mediation is in place if both the association between the variables of interaction quality and mediators yielded in the first step

as well as the association between the mediators and the negative emotional fit yielded in the third step, were significant.

2.1.1 Emotional intensities as mediators of the link between exclusion by the majority interaction partners and minorities' emotional fit in negative interactions.

The first step, in which we regressed each variable denoting emotional intensities separately on *exclusion by the majority interaction partner*, yielded the following significant associations: $\beta = .351$, $p < .001$ for negative autonomy-promoting emotions, and $\beta = .441$, $p < .001$ for negative relatedness-promoting emotions. The second step yielded significant associations between minorities' emotional fit in negative interactions and each of the mediator variables: $\beta = .398$, $p < .001$ for negative autonomy-promoting emotions, $\beta = -.369$, $p < .001$ for positive autonomy-promoting emotions, $\beta = -.122$, $p < .001$ for negative relatedness-promoting emotions, and $\beta = -.212$, $p < .001$ for positive relatedness-promoting emotions. The third step analyses, in which we entered the control variables, the variable *exclusion by the majority interaction partner* and the mediators in separate regression models, showed that intensities of negative autonomy-promoting emotions and negative-relatedness promoting emotions mediated the link between exclusion and emotional fit in negative interactions, but their mediation was partial since the main association remained significant (see Mediation models 1 - 2 on Table 1). Yet, the most distinctive mediation was offered by the autonomy-promoting emotions: only when considering the mean intensities of emotions such as frustration, anger, and disappointment, exclusion by the majority interaction partner and emotional fit in negative interactions were no longer positively but negatively associated.

2.1.2 Emotional intensities as mediators of the link between acceptance by the majority interaction partner and minorities' emotional fit in negative interactions.

The first step, in which we regressed each emotion type variable separately on *acceptance by the majority interaction partner*, yielded the following significant associations: $\beta = -.288$, $p < .001$ for negative autonomy-promoting emotions, $\beta = .132$, $p < .01$ for positive autonomy-promoting emotions, $\beta = .218$, $p < .001$ for negative relatedness-promoting emotions, $\beta = .830$, $p < .001$ for positive relatedness-promoting emotions. As shown above, the second step yielded significant associations between emotional fit in negative interactions and each of the mediator variables. The third step analyses, in which we entered the control variables, the variable *acceptance by the majority interaction partner* and each of the mediators in separate regression models, showed that intensities of all types of emotions mediated the link between acceptance by the majority interaction partner and emotional fit in negative interactions. Yet their mediation was partial since the association between feeling accepted and emotional fit remained significant and negative (see Mediation models 1 - 4 in Table 2).

2.1.3 Emotional intensities as mediators of the link between 'the majority interaction partner is different' and minorities' emotional fit in negative interactions.

The first step, in which we regressed each emotion type variable separately on *majority partner is different*, yielded the following significant associations: $\beta = .261$, $p < .001$ for negative autonomy-promoting emotions, and $\beta = -.411$, $p < .001$ for positive relatedness-promoting emotions. The third step analyses, in which we entered the control variables, the variable *majority partner is different* and each of the mediators in separate regression analyses, showed that negative autonomy-promoting emotions and positive relatedness-promoting emotions fully mediated the link between seeing the majority interaction partner as different and emotional fit in negative interactions (see Mediation models 1 - 2 on Table 3).

2.1.4 Emotional intensities as mediators of the link between strength of relationship and minorities' emotional fit in negative interactions.

The first step, in which we regressed each emotion type variable separately on *relationship strength*, yielded the following significant associations: $\beta = .186$, $p < .001$ for negative relatedness-promoting emotions, and $\beta = .555$, $p < .001$ for positive relatedness-promoting emotions. The third step analyses, in which we entered the control variables, the variable *relationship strength* and each mediator in separate regression models, showed that the emotional intensities variables partially mediated the link between strength of relationship with the majority interaction partner and minorities' emotional fit in negative interactions (see Mediation models 1 – 2 on Table 4). Yet, the most distinctive mediation was offered by the positive relatedness-promoting emotions: only when considering the mean intensities of emotions such as closeness, trust, and respect, the strength of the relationship with the interaction partner and emotional fit in negative interactions were no longer negatively but positively associated.

Table 1. Minorities' emotional fit by mean emotion intensities and exclusion by the majority interaction partner.

	Emotional fit by exclusion by the majority partner	Mediation model 1	Mediation model 2
Fixed Part			
Intercept	0.659 (0.080)***	0.606 (0.052)***	0.659 (0.077)***
Age	-0.039 (0.035)	-0.015 (0.023)	-0.061 (0.034)
Gender	0.036 (0.082)	0.004 (0.055)	0.049 (0.080)
Frequency of interaction	-0.181 (0.062)**	-0.139 (0.039)***	-0.224 (0.059)***
Length of acquaintance	0.068 (0.063)	0.021 (0.039)	0.069 (0.060)
Exclusion by the majority partner	0.095 (0.032)**	-0.045 (0.021)*	0.170 (0.033)***
Negative autonomy-promoting emotions		0.412 (0.020)***	
Negative relatedness-promoting emotions			-0.190 (0.033)***
Random Part			
Level: Participant	0.070 (0.021)	0.037 (0.009)	0.071 (0.020)
Level: Interaction	0.175 (0.018)	0.064 (0.007)	0.153 (0.016)
-2*loglikelihood:	371.587	110.178	340.311
Units: Participant	90	90	90
Units: Interaction	277	277	277

The mediation models were regression models where together with the control variables and the variable *exclusion by the majority interaction partner*, the variables denoting mean intensities of negative autonomy-promoting emotions (frustration anger disappointment; Model 1) and negative relatedness-promoting emotions (guilt, shame, indebtedness; Model 2) were included.
Note. $^{*}p < .05$, $^{**}p < .01$, $^{***}p < .001$ (2-tailed)

Table 2. Minorities' emotional fit by mean emotion intensities and acceptance by the majority interaction partner.

	Emotional fit by acceptance by the majority partner	Mediation model 1	Mediation model 2	Mediation model 3	Mediation model 4
Fixed Part					
Intercept	0.621 (0.073)***	0.594 (0.049)***	0.581 (0.066)***	0.622 (0.074)***	0.574 (0.074)***
Age	-0.042 (0.032)	-0.014 (0.022)	-0.035 (0.029)	-0.054 (0.033)	-0.041 (0.032)
Gender	0.027 (0.075)	0.008 (0.051)	-0.000 (0.067)	0.030 (0.076)	0.042 (0.074)
Frequency of interaction	-0.114 (0.059)	-0.107 (0.038)***	-0.070 (0.053)	-0.140 (0.059)**	-0.071 (0.059)
Length of acquaintance	0.080 (0.059)	0.025 (0.038)	0.075 (0.053)	0.086 (0.058)	0.091 (0.058)
Acceptance by the majority partner	-0.212 (0.029)***	-0.104 (0.020)***	-0.164 (0.027)***	-0.194 (0.030)***	-0.087 (0.044)*
Negative autonomy-promoting emotions		0.370 (0.019)***			
Positive autonomy-promoting emotions			-0.321 (0.038)***		
Negative relatedness-promoting emotions				-0.078 (0.030)*	
Positive relatedness-promoting emotions					-0.151 (0.041)***
Random Part					
Level: Participant	0.055 (0.017)	0.030 (0.008)	0.046 (0.014)	0.061 (0.018)	0.057 (0.017)
Level: Interaction	0.155 (0.016)	0.061 (0.006)	0.123 (0.012)	0.149 (0.015)	0.146 (0.015)
-2*loglikelihood:	333.035	88.501	270.424	326.691	320.045
Units: Participant	90	90	90	90	90
Units: Interaction	277	277	277	277	277

The mediation models were regression models where together with the control variables and the variable *acceptance by the majority interaction partner*, the variables denoting mean intensities of different types of emotions were included: such as negative autonomy-promoting emotions (frustration anger disappointment; Model 1), positive autonomy-promoting emotions (happiness, pride, elation; Model 2), negative relatedness-promoting emotions (guilt, shame, indebtedness; Model 3), and positive relatedness-promoting emotions (closeness, trust, respect; Model 4).

Note. $^{*}p < .05$, $^{**}p < .01$, $^{***}p < .001$ (2-tailed)

Table 3. Minorities' emotional fit by mean emotion intensities and 'perceiving majority interaction partner as different from oneself'.

	Emotional fit by 'majority partner is different'	Mediation model 1	Mediation model 2
Fixed Part			
Intercept	0.653 (0.079)***	-0.557 (0.081)***	1.011 (0.093)***
Age	-0.041 (0.035)	-0.013 (0.024)	-0.040 (0.032)
Gender	-0.009 (0.082)	0.005 (0.057)	0.036 (0.076)
Frequency of interaction	-0.167 (0.061)***	-0.139 (0.039)***	-0.070 (0.059)
Length of acquaintance	0.094 (0.062)	0.020 (0.040)	0.097 (0.058)
Majority partner is different	0.112 (0.024)***	0.011 (0.016)	0.032 (0.026)
Negative autonomy-promoting emotions		0.394 (0.020)***	
Positive relatedness-promoting emotions			-0.193 (0.031)***
Random Part			
Level: Participant	0.073 (0.021)	0.040 (0.010)	0.061 (0.018)
Level: Interaction	0.165 (0.017)	0.064 (0.007)	0.146 (0.015)
-2*loglikelihood:	359.205	114.328	322.266
Units: Participant	90	90	90
Units: Interaction	277	277	277

The mediation models were regression models where together with the control variables and the variable *majority interaction partner is different*, the variables denoting mean intensities negative autonomy-promoting emotions (frustration anger disappointment; Model 1) and positive relatedness-promoting emotions (closeness, trust, respect; Model 2) were included.

Note. $^{***}p < .001$ (2-tailed)

Table 4. Minorities' emotional fit by mean emotion intensities and relationship strength.

	Emotional fit by relationship strength	Mediation model 1	Mediation model 2
Fixed Part			
Intercept	0.570 0.084)***	0.591 (0.084)***	0.620 (0.078)***
Age	-0.048 (0.034)	-0.062 (0.035)	-0.037 (0.032)
Gender	0.036 (0.081)	0.037 (0.081)	0.047 (0.076)
Frequency of interaction	-0.100 (0.068)	-0.150 (0.068)**	-0.113 (0.062)
Length of acquaintance	0.110 (0.065)	0.108 (0.063)	0.067 (0.059)
Relationship strength	-0076 (0.026)***	-0.056 (0.026)*	0.071 (0.030)**
Negative relatedness-promoting emotions		-0.105 (0.032)***	
Positive relatedness-promoting emotions			-0.263 (0.034)***
Random Part			
Level: Participant	0.066 (0.020)	0.071 (0.020)	0.063 (0.018)
Level: Interaction	0.178 (0.018)	0.168 (0.017)	0.141 (0.014)
-2*loglikelihood:	371.881	361.677	318.410
Units: Participant	90	90	90
Units: Interaction	277	277	277

The mediation models were regression models where together with the control variables and the variable *relationship strength*, the variables denoting mean intensities of negative relatedness-promoting emotions (guilt, shame, indebtedness; Model 1), and positive relatedness-promoting emotions (closeness, trust, respect; Model 2) were included.

Note. $^{*}p < .05$, $^{**}p < .01$, $^{***}p < .001$ (2-tailed)

2.2 Do minorities experience different levels of emotional intensities from their majority peers?

To explore this question, we conducted a series of multilevel regression analyses with both minority and majority data, focusing only on negative interactions that were characterized by high quality of interaction. Again, we classified the emotion items in four different categories: negative autonomy-promoting emotions (anger, frustration and disappointment), positive autonomy-promoting emotions (happiness, elation and pride), negative relatedness-promoting emotions (shame, guilt and indebtedness),and positive relatedness-promoting emotions (closeness, trust and respect) and computed four new variables denoting the mean intensity of each category. We defined the group of high quality interactions by conducting a median split on each interaction quality variable and choosing the interactions in the high quality interaction group. In separate regression models, we included the majority status variable as a predictor of participants' intensity of each group of emotion. The analyses showed that in high quality negative interactions, minority and majority youth did not differ in their mean emotional intensities (See Tables 5 – 8).

Table 5. The mean intensity of different types of emotions by majority status (data from the negative interactions coded as 'low exclusion by the majority interaction partner')

	Negative autonomy-promoting emotions	Positive autonomy-promoting emotions	Negative relatedness-promoting emotions	Positive relatedness-promoting emotions
Fixed Part				
Intercept	3.059 (0.158)***	1.491 (0.116) ***	1.688 (0.124)***	1.822 (0.162)***
Age	-0.092 (0.065)	0.010 (0.049)	-0.105 (0.051)	0.043 (0.067)
Gender	0.020 (0.141)	-0.061 (0.106)	0.028 (0.111)	-0.001 (0.145)
Frequency of interaction	-0.046 (0.108)	0.146 (0.073)	-0.043 (0.084)	0.563 (0.109)***
Length of acquaintance	-0.016 (0.113)	0.107 (0.077)	-0.041 (0.087)	0.083 (0.114)
Majority status	-0.261 (0.139)	0.114 (0.105)	0.073 (0.109)	0.035 (0.143)
Random Part				
Level: Participant	0.273 (0.074)	0.210 (0.042)	0.182 (0.046)	0.316 (0.079)
Level: Interaction	0.721 (0.068)	0.288 (0.027)	0.417 (0.039)	0.7050.067)
-2*loglikelihood:	994.549	714.488	807.515	998.258
Units: Participant	147	147	147	147
Units: Interaction	360	360	360	360

Note. $^{***}p < .001$ (2-tailed)

Table 6. The mean intensity of different types of emotions by majority status (data from the negative interactions coded as 'high acceptance by majority interaction partner')

	Negative autonomy-promoting emotions	Positive autonomy-promoting emotions	Negative relatedness-promoting emotions	Positive relatedness-promoting emotions
Fixed Part				
Intercept	3.029 (0.158)***	1.728 (0.118)***	2.274 (0.162)***	2.562 (0.139)***
Age	-0.030 (0.065)	0.065 (0.049)	-0.118 (0.067)	0.011 (0.056)
Gender	-0.040 (0.139)	-0.110 (0.105)	0.074 (0.145)	0.154 (0.121)
Frequency of interaction	-0.038 (0.108)	0.023 (0.078)	-0.056 (0.106)	0.463 (0.100)***
Length of acquaintance	-0.024 (0.116)	0.174 (0.084)	-0.162 (0.114)	0.024 (0.107)
Majority status	-0.003 (0.140	0.066 (0.106)	0.142 (0.147)	-0.179 (0.122)
Random Part				
Level: Participant	0.3130.076	0.197 (0.043)	0.390 (0.082)	0.200 (0.057)
Level: Interaction	0.6190.061	0.309 (0.031)	0.558 (0.055)	0.558 (0.055)
-2*loglikelihood:	899.910	683.449	889.703	842.474
Units: Participant	140	140	140	140
Units: Interaction	337	337	337	337

Note. $^{***}p < .001$ (2-tailed)

Table 7. The mean intensity of different types of emotions by majority status (data from the negative interactions coded as 'majority interaction partner is not very different from oneself')

	Negative autonomy-promoting emotions	Positive autonomy-promoting emotions	Negative relatedness-promoting emotions	Positive relatedness-promoting emotions
Fixed Part				
Intercept	3.124 (0.153)***	1.505 (0.109)***	2.142 (0.155)***	2.281 (0.152)***
Age	-0.107 (0.063)	0.042 (0.046)	-0.145 (0.065)**	0.004 (0.063)
Gender	-0.075 (0.134)	-0.088 (0.098)	-0.011 (0.138)	0.025 (0.134)
Frequency of interaction	0.050 (0.105)	0.140 (0.068)	0.009 (0.102)	0.514 (0.102)***
Length of acquaintance	-0.116 (0.110)	0.124 (0.071)	-0.133 (0.107)	0.037 (0.107)
Majority status	-0.058 (0.137)	0.099 (0.100)	0.092 (0.141)	-0.109 (0.137)
Random Part				
Level: Participant	0.309 (0.074)	0.217 (0.040)	0.380 (0.079)	0.339 (0.075)
Level: Interaction	0.706 (0.064)	0.263 (0.024)	0.625 (0.057)	0.641 (0.058)
-2*loglikelihood:	1080.170	750.840	1059.635	1057.801
Units: Participant	159	159	159	159
Units: Interaction	390	390	390	390

Note. $^{**}p < .01$, $^{***}p < .001$ (2-tailed)

Table 8. The mean intensity of different types of emotions by majority status (data from the negative interactions coded as 'high relationship strength')

	Negative autonomy-promoting emotions	Positive autonomy-promoting emotions	Negative relatedness-promoting emotions	Positive relatedness-promoting emotions
Fixed Part				
Intercept	3.250 (0.296)***	1.305 (0.194)***	2.647 (0.261)***	3.092 (0.283)***
Age	-0.061 (0.095)	0.068 (0.061)	-0.138 (0.081)	0.112 (0.091)
Gender	0.155 (0.194)	-0.087 (0.124)	-0.181 (0.165)	0.015 (0.184)
Frequency of interaction	-0.127 (0.215)	0.329 (0.144)	-0.094 (0.195)	0.185 (0.206)
Length of acquaintance	-0.099 (0.202)	0.150 (0.137)	-0.413 (0.189)*	-0.285 (0.194)
Majority status	0.044 (0.198)	0.045 (0.127)	0.243 (0.168)	-0.282 (0.188)
Random Part				
Level: Participant	0.530 (0.125)	0.180 (0.052)	0.265 (0.092)	0.464 (0.113)
Level: Interaction	0.635 (0.081)	0.332 (0.042)	0.690 (0.086)	0.602 (0.077)
-2*loglikelihood:	659.088	482.806	634.712	641.628
Units: Participant	114	114	114	114
Units: Interaction	232	232	232	232

Note. $^{*}p = .05$, $^{***}p < .001$ (2-tailed)
